# *In vivo* analysis of puerarin from *Pueraria tuberosa* as a promising galactagogue

**DOI:** 10.6026/9732063002001574

**Published:** 2024-11-30

**Authors:** Kiran R. Giri, Kamlesh M Palandurkar, Ved Prakash, Amit Singh, Anshuman Trigunayat

**Affiliations:** 1 Department of Pharmacology, Institute of Medical Sciences, Banaras Hindu University, Varanasi - 221005, Uttar Pradesh, India; 2 Department of Biochemistry, Institute of Medical Sciences, Banaras Hindu University, Varanasi - 221005, Uttar Pradesh, India; 3 Department of Biochemistry, Nandkumar Singh Chouhan Government Medical College, Khandwa, Madhya Pradesh, India

**Keywords:** Galactagogue, breastfeeding, *Pueraria tuberosa*, puerarin, aquaporin channel

## Abstract

Breastfeeding is crucial for maternal and infant health, yet global rates vary, with India having the highest at 43.2% and the lowest at 35.8%.To evaluate the galactagogue properties of *Pueraria tuberosa* in a female rat model, assess its safety through acute toxicity studies and examine its impact on hormone regulation. Secondary objectives included analyzing its effect on serum prolactin levels and conducting molecular docking and GC-MS analyses. Ethical approval was obtained (Letter No. Dean/2019/IEAC/1614). Charles Foster rats were housed under standard conditions and divided into four groups: vehicle, *Pueraria tuberosa* extracts, 15 mg/kg/day of Puerarin in DMSO and saline and from day three today fifteen of parturition, 2.7 mg/kg of domperidone. Weekly body weights and milk yield were recorded. Serum prolactin levels were measured using the Rat Prolactin ELISA Kit. GC-MS analysis identified *Pueraria tuberosa* extract constituents. Imaging with the Vevo LAZR System evaluated breast areas and molecular docking studies targeted the 5-HT2AR and D2 receptors. Significant differences in milk yield were observed between the 3rd and 14th days across treatment groups, with Puerarin and domperidone significantly increasing serum prolactin levels. Imaging indicated increased breast blood flow in Puerarin-treated rats, suggesting enhanced lactation. Molecular docking showed high affinity of Puerarin for D2 and 5HT2A receptors, indicating potential hormonal regulation mechanisms. This research provides a comprehensive preclinical evaluation of Puerarin's efficacy and safety as a galactagogue, addressing gaps in understanding herbal lactation aids and laying the foundation for future clinical trials and comparisons with established galactagogues.

## Background:

Breastfeeding is the best way to feed new-borns and has many health advantages for moms and babies. Breastfeeding improves mother and infant health, according to a compilation of systematic reviews and meta-analyses by the Agency for Healthcare Research and Quality (AHRQ) [1]. Infants who are not breastfed face elevated risks of various health issues, including diarrhea, lower respiratory tract infections even may lead to sudden death syndrome. Moreover and breast feeding provides protection against breast and ovarian cancers for mothers [2]. Despite the well-established benefits of breastfeeding, the global landscape reveals variations in breastfeeding rates. In the Indian context, a report titled "Exclusive Breastfeeding in India: Trends and Data Gaps" by the International Food Policy Research Institute (IFPRI) highlighted an exclusive breastfeeding rates, with the highest at 43.2% and the lowest at 35.8%. The report further indicated that the incidence rates of breastfeeding in India from 2015 to 2021 were 48% within the first hour after birth, 44% at six months and 68% and 44% at one and two years, respectively [3]. *Pueraria tuberosa* (Indian Kudzu) is a perennial herb with extensive pharmacological potential, containing compounds like puerarin and genistein [4]. This is first report on *Pueraria tuberosa* and its active component Puerarin as galactagogue focusing on Aquaporin channel expression and Photoacoustic study. Therefore, it is of interest to report an *In vivo* analysis of puerarin from *Pueraria tuberosa* as a promising galactagogue.

The primary objectives of this study focus on assessing the galactagogue properties and safety of *Pueraria tuberosa*. To evaluate its efficacy in enhancing lactation, an experimental screening will be conducted using a female rat model. Additionally, the safety of *Pueraria tuberosa* will be assessed through acute toxicity studies to identify any immediate adverse effects. Secondary objectives include investigating the herb's influence on hormonal regulation, particularly its impact on serum prolactin levels, which are critical for lactation. The study also involves molecular and chemical characterization; molecular docking analysis will explore interactions between bioactive compounds, including puerarin and lactation-related receptors, offering insights into potential molecular mechanisms. Further, GC-MS analysis will be employed to identify and quantify the chemical constituents of *P. tuberosa*, providing a detailed understanding of its composition and bioactive potential.

## Materials and Methods:

## Animals:

After receiving the ethical approval letter no. Dean/2019/IEAC/1614 dated 17.11.2019; Charles Foster rats were procured from IMS, BHU and weighed at the beginning of the study. Animals, after five days of acclimatization, were randomly grouped and marked. Animals were housed separately in cages. The animals were kept under standard laboratory conditions with a 12-hour light: 12-hour dark cycle and a 25±2°C temperature throughout the study. Ad libitum tap water was given to the rats throughout the experiment, along with standard rat diet.

## Grouping of animals:

**Figure 1:**
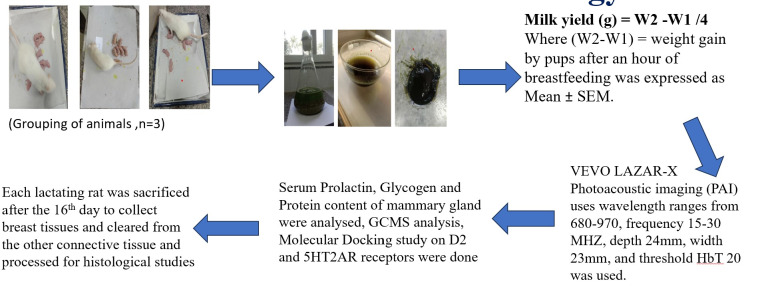
Overall schematic scheme of paper

Animals were assigned to each of four groups, with six mothers and six or seven pups per group. (Figure 1) Normal control rats in group 1 received vehicle orally, given as 2 ml of 1% carboxymethyl cellulose sodium in normal saline. The dose of 150 mg/kg/day of *Pueraria tuberosa* aqueous extracts were given to Group 2, while Group 3 administered 15mg/kg/day of Puerarin (82425-Sigma Aldrich) dissolved in 2% DMSO and diluted with saline and Group 4 was given 2.7 mg/kg body weight of domperidone suspended in vehicle from the 3rd to the 15th day post-partum [5].

## Toxicity study:

During the initial stage, 300 mg/kg body weight was administered to 3 female animals. A second stage, according to the OECD guideline 423, another set of three received 2000 mg/kg body weight after no mortality. The final phase was giving another batch of three female animals' 2000 mg/kg body weight, which did not produce any mortality. The experimental procedure involved the single oral administration of the substance through gavage. Prior to administration, a fasting period of at least 12 hours was implemented, with access to water available ad libitum. The substance was administered in the morning and the observation period extended for 14 days. Body weights were measured individually shortly before administration (day 0), daily thereafter and on the final day of observation. Clinical observations were conducted and signs were recorded for each animal on the day of administration and at least once daily. Mortality checks were performed to identify any dead or moribund animals throughout the experimental period [6].

## Milk yield:

In this experiment, rat pups were weighed at different time points after their mother received treatment. Initially, pups were weighed 13 hours post-treatment (w1), then isolated and weighed again after 4 hours (w2). (Figure 1) Following this, they were reunited with their mother for 1 hour, weighed (w3), isolated again for 4 hours, weighed (w4), reunited for another hour and weighed once more (w5). Overnight, they remained with their mother. Milk yield measurements were derived from these weights, with the estimate at 18 hours post-treatment calculated as (w3 - w2) and at 23 hours as (w5 - w4). To correct for pup weight loss during suckling, a correction factor was determined as (w2 - w1)/4 (weight loss per hour during suckling) and applied to the daily milk yield at 18 hours, considering suckling hours. Pups' daily weight increase was computed from their weight at w2 and the mother rat's weight was checked daily, with the difference between weights on the 3rd and 15th days following parturition recorded [7] (Table 1).

## Estimation of the glycogen and protein content mammary gland tissue:

## Mammary gland tissue glycogen and protein estimation:

On day 16, mother rats were sacrificed and blood samples taken. The mammary gland was removed from connective tissue and weighed using an electronic scale. To homogenise 100 mg of tissue, mix 1.5 ml of distilled water homogeniser with 1.5 ml of 30% KOH saturated with Na2SO4, heat in a boiling water bath for 30 minutes, cool and centrifuge after adding 2 ml of 95% ethanol. Glycogen precipitated after alkaline digestion was dissolved in distilled water and quantified using phenol-sulphuric acid. Total protein kit was used to analyse protein content (Table 2).

## Prolactin estimation by ELISA:

Rat Prolactin ELISA Kit (ab272780) was used for the quantitative determination of prolactin antigen in rat serum (Table 3).

## Gas chromatography:

GC-MS analysis was performed using the GCMS-QP-2010 plus equipment from Shimadzu Co., Kyoto, Japan. The injection settings were split injection with a 10:1 ratio and 260°C injection temperature. The chosen column was Rxi-5 SIL MS column with dimensions of 30 M x 0.25 mm id x 0.25 μm film thickness. Mass spectrometer adjustment followed manufacturer instructions for best performance. The interface temperature was maintained at 270°C and the ion source at 220°C. Helium flows 1.21 ml min-1 as the carrier gas. The temperature regimen began with 2 minutes of isothermal heating at 60°C and then increased to 250°C for 2 minutes and 280°C for 21 minutes. Thermo Quest (Manchester, UK) was used to analyze chromatograms and mass spectra. Automatic peak measurement of metabolite derivatives in mass lab methods was made possible by retention time and mass spectral library (Table 5).

## Photoacoustic activity:

**Figure 2:**
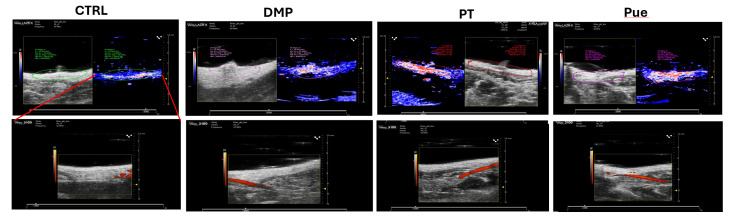
Vevo LAZR imaging system for micro ultrasound and photoacoustic imaging in lactating rats.

Imaging and quantification were done using the Vevo LAZR Imaging System (FUJIFILM Visual Sonics, Inc.). This platform integrates micro ultrasound and photoacoustic imaging. In a linear-array ultrasonic transducer (LZ250, fc = 21 MHz; LZ550, fc = 50 MHz), fibre optic bundles transmitted light from a tunable laser (680-970 nm). The rats were scanned under isoflurane. The active breast portions of the rats were marked for assessment and each breast are was maintained the same size for comparison (Figure 2).

## Molecular docking study:

The compounds selected as ligands for this study, including anethole [PMID 637563], pentanone [PMID 7895], lepidiline A [PMID 404702], lepidine [PMID 10285], lepidine E [PMID 100927767], Puerarin [PMID 5281807], daidzein [PMID 5281708], shatavarin IV [PMID 441896], sparsasapogenin, trigonelline, vitexin, cocrystallized ligand (8NU) and risperidone (as a control), were chosen based on their known activity. The 3D chemical structures of these ligands were retrieved from the PubChem database and CID 5073 was assigned to risperidone. The ligands were initially obtained in .sdf format from PubChem and then converted into Protein Data Bank (PDB) format using PyMoL software. The target proteins for the molecular docking study were 5-hydroxytryptamine 2A receptor (5-HT2AR) and prolactin receptor (PRLR) and their respective 3D structures were obtained from the Protein Data Bank with the PDB accession IDs 6A93 and 3D48. PyMoL software was utilized not only for converting ligands and proteins into PDB format but also for the removal of water molecules from both ligands and target proteins. The data sources for ligand structures were the PubChem database (https://pubchem.ncbi.nlm.nih.gov/), while the Protein Data Bank (https://www.rcsb.org/) provided the protein structures. Risperidone served as the control ligand in the study. The 3D structures of both ligands and proteins are crucial for understanding interactions in molecular docking studies. The proper format conversion and removal of water molecules, as achieved using PyMoL, contributed to the accuracy of the structural conformation in this research [8, 9, 10- 11] (Table 5).

## Histopathology:

In this study, lactating rats were utilized as subjects, with each rat being sacrificed on the 16th day of lactation. Breast tissue was collected for histopathological studies. Breast tissue was cleared from other connective tissues. Fixation was carried out by immersing the breast tissues in a 10% formalin solution. The microscopic analysis aimed to provide insights into the histopathological features of the breast tissues. Specifically, the parenchyma area of the breast tissues was meticulously compared to the mammary stroma (Figure 4).

## RT-PCR Methodology:

This study focused on gene expression analysis of aquaporin 1, aquaporin 3, aquaporin 5 and aquaporin 7, with the housekeeping gene GAPDH, in rats. Aquaporins contribute to the transport of water, affecting the overall composition of breast milk. AQP1, AQP3 and AQP7 are involved in the transport of water, influencing the concentration of milk constituents. The primer sequences for these genes were obtained from the NCBI database and further validated through Primer-BLAST. Subsequently, in silico PCR was conducted to check the specificity of the primers. Following validation, individual reactions were carried out using ExicyclerTM96 RealTime Quantitative Thermal Block by Bioneer. After 5 minutes of reverse transcription at 42°C, enzyme activation at 95°C took 3 minutes. Amplification included denaturation at 95°C for 1-3 seconds and elongation at 60°C for 20 seconds. The cycle threshold (Ct) value determined gene expression levels using the formula: CT fold change = 2^ (-ΔΔCT). The ΔCT was calculated by subtracting the housekeeping gene GAPDH Ct value from the target gene (AQP1, AQP3, AQP5, or AQP7) Ct value. Moreover, ΔΔCT was computed by subtracting the treatment group's ΔCT from the control groups. The purpose was to compare aquaporin gene expression to GAPDH. Primers were selected to match annealing temperatures, allowing multiplexing all four genes using GAPDH as the housekeeping gene. This method allows for a thorough gene expression study, revealing aquaporins' significance in the rat model [12] (Figure 5).

Aquaporin 1 (Forward TGCCGTTAACCATGTCGTGA),

Reverse (GTCCAGGCAGAAACGGAGAA),

Aquaporin 3 (Forward TCTCCAGGCTGAAAGCAAGG)

Reverse (TCCACACTGGAGTCCCTGAA)

Aquaporin 5 (Forward CCAGAAAGGGACGACAGCTT)

Reverse (GTGGTTTATTGGGAAGCGCC)

## GAPDH:

Forward (TGTGAACGGATTTGGCCGTA)

Reverse (GATGGTGATGGGTTTCCCGT)

## Statistical analysis:

The data were presented as Mean ± SEM and a Post Hoc test was conducted to assess intergroup differences.

## Results:

Table 1 Comparison of milk yield (my) in different treatment groups over fourteen days the p values (3rd vs 14th day) indicate the statistical significance of the difference in milk yield between the 3rd and 14th days for each treatment group, with values less than 0.001 considered highly significant. Values are presented as mean ± standard deviation. Significant differences are denoted by *** with corresponding P values. Values less than 0.05 are considered statistically significant. The Table 3 compares serum prolactin levels (ng/ml) in various treatment groups, including Control, Puerarin, Domperidone and Aqueous extract of *Pueraria tuberosa* (PT). Values are presented as mean ± standard deviation. Significant differences are ** Values less than 0.05 are considered statistically significant. The p-values obtained from Tukey's HSD test are adjusted for multiple comparisons to reduce the chance of Type I errors (false positives) when conducting multiple pairwise comparisons. The Table 5 shows the compounds obtained from the GC-MS analysis of *Pueraria tuberosa* extract. CTRL: Control, DMP: Domperidone, PT: *Pueraria tuberosa*, Pue: Puerarin. Figure 2 High contrasts and resolution photoacoustic imaging (PAI) detects blood haemoglobin (HB) concentration and oxygenation (sO2). The above panel image is indicating hemoconcentration and below panel image is indicating oxygen concentration.

The Table 6 presents the docking scores (in kcal/mol) of various ligands with dopamine D2 and serotonin 5-HT2A receptors. Lower docking scores indicate stronger binding affinity. Ligands include compounds like Anethole, Lepidiline A, Puerarin and others, along with a co crystallized ligand (8NU) used as a reference. The figure illustrates the relative gene expression levels of aquaporin 1 (AQP1), aquaporin 3 (AQP3), aquaporin 5 (AQP5) and aquaporin 7 (AQP7) in a rat model. The gene expression analysis was conducted using validated primer sequences, multiplexing all four genes while utilizing GAPDH as the housekeeping gene.

## Discussion:

In comparison with the literature, the findings of the study on Puerarin, a potential galactagogue derived from *Pueraria tuberosa*, present a comprehensive evaluation of its efficacy and safety. This research extensively addresses the lacunae in current knowledge regarding herbal galactagogues. Safety evaluation through acute toxicity studies conform to established guidelines, ensuring accurate assessment effects of the compound. This approach aligns with the recommendations made by Meier and Engstrom (2007) for assessing pharmacologic agents affecting milk production. This study further explored hormonal regulation by measuring the serum prolactin concentration, shedding light on the impact of Puerarin on key lactation-related hormones. The results show that both Puerarin and Domperidone significantly increased serum prolactin levels compared to the control group, with Puerarin having the highest elevation. Interestingly, the aqueous extract of *Pueraria tuberosa* (PT) did significantly alter serum prolactin levels compared to the control. Such an in-depth investigation is crucial given the limited clinical evidence available, as highlighted by Zheng *et al.* (2018), emphasizing the need for rigorous scientific evaluation of herbal galactagogues [13].

**Figure 3:**
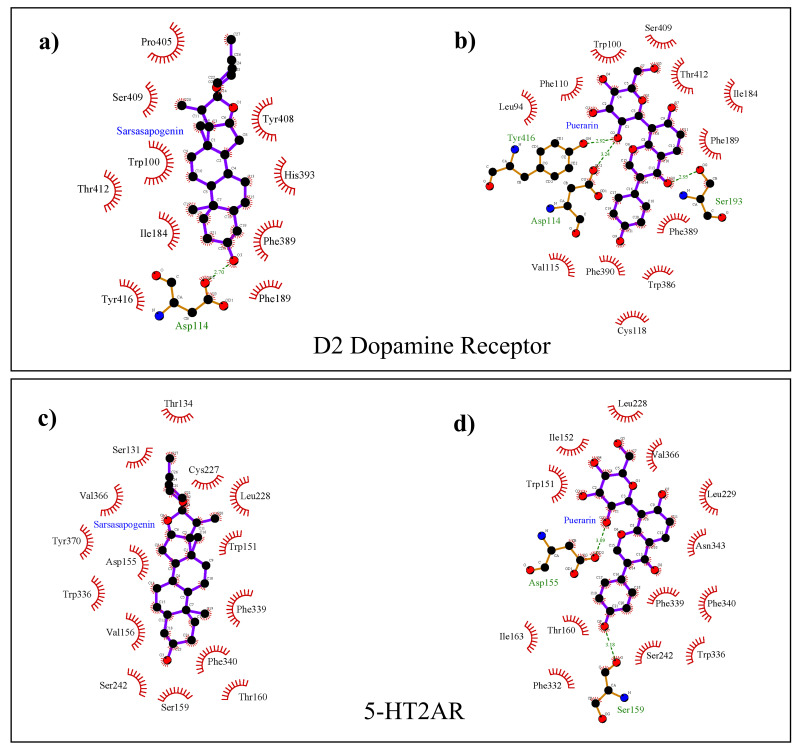
Docking ligands with dopamine D2 and Serotonin 5-HT2A receptors of different bioactive compounds

**Figure 4:**
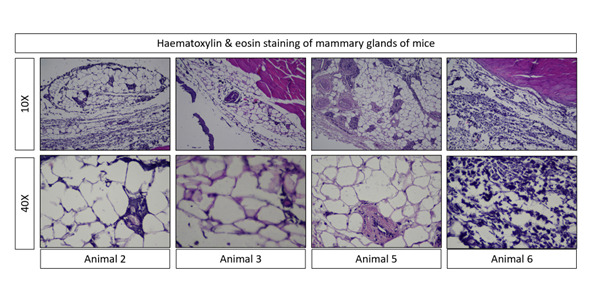
Histopathological comparison of parenchyma and mammary stroma in lactating rat breast tissues at 10x magnification

**Figure 5:**
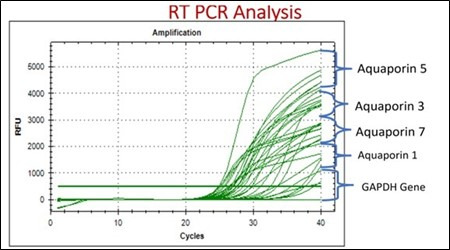
Relative gene expression levels of aquaporins in rat model.

Aquaporins play a vital role in breast milk secretion by facilitating the transport of water and other essential nutrients across mammary gland cells. Their presence ensures efficient hydration and nutrient delivery, crucial for maintaining milk volume and composition during lactation. Dysregulation of aquaporin function can impact milk production and quality, highlighting their significance in the physiology of lactation. The relative gene expression levels of aquaporin 1 (AQP1), aquaporin 3 (AQP3), aquaporin 5 (AQP5) and aquaporin 7 (AQP7) in the rat model provide valuable insights into their roles in water transport and homeostasis. The use of validated primer sequences and multiplexing techniques, along with GAPDH as the housekeeping gene, ensures the accuracy and reliability of the gene expression analysis. The differential expression patterns observed among these aquaporins may indicate their specific contributions to water movement in different tissues or physiological conditions, highlighting their importance in maintaining cellular hydration and function in the rat model. The Vevo LAZR Imaging System ensured consistent breast area measurements in rats, crucial for comparative analysis. Puerarin-treated rats displayed increased blood flow in active breast areas, suggesting a potential mechanism for its lactation-enhancing effects, as seen in advanced imaging. This observation offers valuable insights into Puerarin's pharmacological impact on milk production. This molecular docking study, which revealed the affinity of Puerarin for the D2 and 5HT2A receptors, provides a valuable mechanistic perspective that is correlated with hormonal regulation. Notably, compounds like Puerarin (-9.7 for D2, -9.6 for 5-HT2AR), Sarsasapogenin (-10.2 for D2, -11.1 for 5-HT2AR) and the co-crystallized ligand (8NU) (-12.0 for D2, -11.3 for 5-HT2AR) exhibited particularly high affinity for both receptors, suggesting their potential as effective ligands for pharmacological interactions. (Figure 3) Dopamine D2 receptors inhibit prolactin release, affecting milk production, while serotonin 5-HT2A receptors modulate neural signals, influencing oxytocin release for milk ejection during breastfeeding. Both receptors play crucial roles in regulating hormonal and neural processes essential for successful lactation. This finding aligns with existing research by Marshall, Hernandez (2014) showing the significance of understanding molecular interactions for potential galactagogues [14].

Additionally, histo-pathological examination provides visual confirmation of the activity of the compound at the tissue level, complementing the biochemical and molecular findings. These multipronged methodologies enhance the credibility of the study and address the existing gap in mechanisms of action [15]. Compounds identified in the GC-MS analysis of *Pueraria tuberosa*, such as 5-Hydroxymethylfurfural and Puerarin, may enhance lactation by stimulating prolactin secretion and mimicking estrogen's effects. Additionally, Oleic Acid can improve milk quality, while Stigmasterol may modulate hormonal pathways involved in milk synthesis, emphasizing the extract's potential for promoting milk production (Table 4) [16]. The observed increase in milk yield, glycogen and protein content in mammary tissues signifies the presence of galactogenic properties of Puerarin. While Puerarin is derived from *Pueraria tuberosa*, its comparison with other well-known galactagogues, such as fenugreek, fennel and blessed thistle, could be explored in future studies to provide a more inclusive understanding of its relative efficacy. *Pueraria tuberosa* and Puerarin groups showed consistent and potentially enhanced MY compared to the control, especially evident on the 14th day. Moreover, the study acknowledges the limitations and variability in responses, prominence the need for standardized dosages and long-term safety assessments, consistent with the concerns raised by Gogtay, Bhatt. Overall, this study on Puerarin contributes significantly to the field by presenting a detailed preclinical evaluation, laying the groundwork for potential clinical trials and future comparisons with established galactagogues [17]. Cao S, Li X *et al.* study demonstrates that dietary supplementation with puerarin positively impacted the immune response and antioxidant capacity of sows, as evidenced by increased glutathione peroxidase activity and immunoglobulin levels. Furthermore, puerarin supplementation was associated with improved daily body weight gain and mean body weight at weaning in piglets, suggesting potential benefits for offspring development [18]. Wang R, Li T, Pan Z, study's findings highlight the potential of puerarin as a dietary supplement to enhance sow health and performance, with implications for piglet growth and overall herd productivity. The study demonstrates that dietary supplementation of puerarin in pigeons enhances thymus index, serum immunoglobulin levels, antioxidant activities and intestinal morphology, suggesting potential benefits for immune response, antioxidant capacity and gut health in avian production systems [19]. Lyu C, Yuan B *et al.* investigated whether puerarin (PUE), a natural flavonoid, may reduce H2O2-induced oxidative stress and blood milk barrier disruption in bovine mammary epithelial cells (BMECs) in lactating dairy cows. In H2O2-exposed BMECs, PUE administration boosted antioxidant enzyme activity and decreased ROS and MDA levels. PUE also restored oxidative stress and tight junction protein genes, improving cell-cell interactions. It also inhibited H2O2-induced BMEC inflammatory factors. *In vivo* findings supported these results, showing that feeding PUE to lactating dairy cows reduced inflammatory factor expression in milk and serum. This study suggests that PUE could be a promising preventive and therapeutic agent against mastitis and oxidative stress in dairy farming, potentially serving as a beneficial feed additive [20].

## Conclusion:

In conclusion, a comprehensive assessment of Puerarin, an active constituent of *Pueraria tuberosa*, revealed that it is a promising galactagogue. The acute toxicity study indicated no apparent signs of toxicity or adverse effects at various compound doses. Notably, the galactogogue property assessment demonstrated a significant increase in milk yield in the treated groups, accompanied by a rise in daily weight gain of offspring, suggesting a potential benefit for maternal lactation and offspring development. Hormonal regulation assessment points to elevated prolactin levels, implicating Puerarin and Sarsasapogenin in modulating regulators associated with lactation. Molecular docking and photoacoustic analysis provide valuable insights into potential mechanisms of action, supporting the observed physiological effects. Additionally, increased glycogen and protein content in mammary gland tissues, coupled with structural changes in breast tissues revealed through histopathological analysis, further support the positive outcomes. Aligning with the traditional Ayurvedic use of galactagogues and substantiating their safety and efficacy through molecular interactions, Puerarin has emerged as a natural galactagogue candidate. In the future, promising preclinical results lay the groundwork for future clinical trials, which are essential for confirming safety and efficacy and addressing knowledge gaps in dosage standardization and long-term safety for human lactation. In summary, Puerarin holds substantial potential for development as a safe and effective natural galactagogue, warranting further exploration in clinical settings.

## List of abbreviation:

*Pueraria tuberosa*: PT

Control: CTR

Domperidone: DMP

Puerarin: PUE

## Institutional animal ethical committee:

This project is approved by the Institutional Animal Ethical Committee, IMS, BHU, ethical approval number: Dean/2019/IEAC/1614 dated 17.11.2019

## Figures and Tables

**Table 1:** Comparative analysis of milk yield (MY) across experimental days in control and treatment Groups

**Groups**	**MY 3rd day (ml)**	**MY 7th day (ml)**	**MY 11th day (ml)**	**MY 14th day (ml)**
CTRL	0.079±0.3	0.101±0.5	0.0505±0.4	0.183±0.2
DMP	0.147±0.01	0.220±0.03	0.236±0.02	0.388±0.02
PT	0.157±0.001	0.130±0.003	0.274±0.005	0.280±0.003
Pue	0.167±0.001	0.130±0.003	0.242±0.005	0.280±0.003

**Table 2:** Comparison of glycogen and protein content in different treatment groups

**Groups**	**Glycogen Content (mg/100 g)**	**Protein Content (mg/100 mg)**
CTRL	6.3±0.47	6.1±0.37
DMP	8.0±0.3	8.5±0.4
PT	7.5±0.5	7.83±0.68
Pue	9.3±0.5***	9.1±0.068***

**Table 3:** Comparative serum prolactin levels in CTRL, DMP, PT and Pue groups

**Serum Prolactin (ng/ml)|**	**Control**	**Domperidone**	**Aqueous extract of *Pueraria tuberosa* (PT)**	**Puerarin**
	11.8±0.89	15.2±1.1**	14.9±1.2	16.5±0.95***

**Table 4:** Comparative analysis of pups' daily body weight gain (g/pup/day) across treatment groups

**Groups(g/pup/day)**	**Pups body wt gain 3rd day (g/pup/day)**	**Pups body wt gain 7th day(g/pup/day)**	**Pups body wt gain 11th day (g/pup/day)**	**Pups body wt gain 14th day(g/pup/day)**
CTRL	18.90+0.74	21.5+0.85	25.93+0.91 **	29.35+2.18
DMP	19.98+0.7	22.2+1.01	33.64+2.23 **	37.2+3.14
PT	19.48+0.42	21.29+0.43	31.9+1.33	34.6+1.51
Pue	19.85+0.72	24.2+1.13 **	32.8+1.36	34.51+1.60 **

**Table 5:** Gas chromatography-mass spectrometry (GC-MS) analysis of *Pueraria tuberosa* extract

***Pueraria tuberosa***		
**Peak#**	**R.Time**	**Compound Name**
10	8.969	5-Hydroxymethylfurfural
29	18.829	Oleic Acid
41	27.75	Stigmasterol
42	28.413	Puerarin

**Table 6:** Molecular docking scores of different ligands with dopamine D2 and serotonin 5-HT2A receptors

**S No**	**Compound Name**	**Docking Score (kcal/mol)**	
		**D2**	**5-HT2AR**
1	Anethole	-6.5	-6.1
2	2-Pentanone	-4.1	-3.8
3	Lepidiline A	-8.6	-8.9
4	Lepidine	-7.5	-7.4
5	Lepidine E	-8.9	-9.2
6	Puerarin	-9.7	-9.6
7	Daidzein	-9.6	-8.7
8	Shatavarin IV	-8.9	-7.1
9	Sarsasapogenin	-10	-11.1
10	Trigonelline	-5.6	-5.4
11	Vitexin	-8.5	-8.8
13	Co crystallized ligand (8NU)	-12	-11.3
